# Nuclear and Membrane Receptors for Sex Steroids Are Involved in the Regulation of Delta/Serrate/LAG-2 Proteins in Rodent Sertoli Cells

**DOI:** 10.3390/ijms23042284

**Published:** 2022-02-18

**Authors:** Sylwia Lustofin, Alicja Kamińska, Małgorzata Brzoskwinia, Joanna Cyran, Małgorzata Kotula-Balak, Barbara Bilińska, Anna Hejmej

**Affiliations:** 1 Department of EndocrinologyInstitute of Zoology and Biomedical Research, Faculty of Biology, Jagiellonian University in Krakow, 30-387 Krakow, Poland; s.lustofin@doctoral.uj.edu.pl (S.L.); ala.kaminska@uj.edu.pl (A.K.); m.brzoskwinia@doctoral.uj.edu.pl (M.B.); joanna.cyran@doctoral.uj.edu.pl (J.C.); barbara.bilinska@uj.edu.pl (B.B.); 2 Department of Anatomy and Preclinical Sciences, University Centre of Veterinary Medicine JU-UA, University of Agriculture in Krakow, 30-059 Krakow, Poland; malgorzata.kotula-balak@urk.edu.pl

**Keywords:** androgen receptors, estrogen receptors, DSL proteins, Notch signaling, Sertoli cells

## Abstract

Delta/Serrate/LAG-2 (DSL) proteins, which serve as ligands for Notch receptors, mediate direct cell–cell interactions involved in the determination of cell fate and functioning. The present study aimed to explore the role of androgens and estrogens, and their receptors in the regulation of DSL proteins in Sertoli cells. To this end, primary rat Sertoli cells and TM4 Sertoli cell line were treated with either testosterone or 17β-estradiol and antagonists of their receptors. To confirm the role of particular receptors, knockdown experiments were performed. mRNA and protein expressions of Jagged1 (JAG1), Delta-like1 (DLL1), and Delta-like4 (DLL4) were analyzed using RT-qPCR, Western blot, and immunofluorescence. Testosterone caused downregulation of JAG1 and DLL1 expression, acting through membrane androgen receptor ZRT- and Irt-like protein 9 (ZIP9) or nuclear androgen receptor (AR), respectively. DLL4 was stimulated by testosterone in the manner independent of AR and ZIP9 in Sertoli cells. The expression of all studied DSL proteins was upregulated by 17β-estradiol. Estrogen action on JAG1 and DLL1 was mediated chiefly via estrogen receptor α (ERα), while DLL4 was controlled via estrogen receptor β (ERβ) and membrane G-protein-coupled estrogen receptor (GPER). To summarize, the co-operation of nuclear and membrane receptors for sex steroids controls DSL proteins in Sertoli cells, contributing to balanced Notch signaling activity in seminiferous epithelium.

## 1. Introduction

A series of studies performed at the beginning of the 21st century and subsequent research clearly demonstrated that proper androgen/estrogen balance is fundamental for normal male sexual development and function in humans and animals [1,2,3,4,5,6]. This balance is governed primarily by aromatase, which catalyzes the irreversible conversion of androgenic steroids (testosterone and androstenedione), produced by testicular interstitium, into the estrogens (estradiol and estrone), as well as by the expression of nuclear androgen (AR) and estrogen receptors (ERα and ERβ), determining cell response to these hormones [7,8]. Due to a discovery of membrane androgen and estrogen receptors (ZRT- and Irt-like protein 9, ZIP9, and membrane G-protein-coupled estrogen receptor, GPER) and their localization in the male gonad, it has become increasingly apparent that the testicular androgen–estrogen system is more complex than initially thought [9,10,11,12,13]. Variations in the balance of sex steroids or their action are related to testicular disorders and infertility [14,15,16] and are also a feature of ageing [17]. Therefore, it appears important to understand the role that the components of this system play in the regulation of testicular cell function.

Sertoli cells constitute the somatic component of the seminiferous epithelium, which controls male germ cell development by providing structural, nutritional, and regulatory support for spermatogenic cells. Androgens are major regulators of postnatal Sertoli cell physiology and, indirectly, spermatogenesis. Testosterone action is indispensable for Sertoli cell maturation, blood–testis barrier formation and maintenance, germ cell meiosis, their differentiation, and release from seminiferous epithelium [18]. Although the role of estrogens in the seminiferous epithelium is less clear-cut, these hormones are considered important modulators of Sertoli cell proliferation, differentiation, survival, and energy metabolism [19,20]. Rodent postnatal Sertoli cells show a dynamic pattern of the expression of nuclear and membrane androgen and estrogen receptors, thereby enabling a co-ordinated response to hormonal stimulation [12,19,21,22].

Delta/Serrate/LAG-2 (DSL) proteins are type I transmembrane proteins characterized by the presence of an extracellular N-terminal DSL motif and epidermal growth factor (EGF)-like repeats. They serve as ligands for the receptors of Notch family (Notch 1–4) to mediate direct cell–cell interactions involved in the determination of cell fate and functioning. In seminiferous epithelium, the proper activity of the Notch pathway is crucial for the balance between spermatogonia proliferation and differentiation. It controls germ cell fate and survival throughout the spermatogenesis and regulates the expression of tight junction proteins [19,23,24,25,26].

In mammals, five canonical DSL proteins were identified. They are classified on the basis of the presence (Jagged/Serrate; JAG1 and JAG2) or absence (Delta-like; DLL1, DLL3, and DLL4) of a cysteine-rich domain. JAG1, JAG2, and DLL4 possess additional Delta and OSM-11-like (DOS) domain [27]. Upon ligand–receptor binding, the ligand undergoes endocytosis into the signal-sending cell. This creates a force that causes a conformational change and promotes receptor activation (called *trans*-activation). Then Notch receptor undergoes two proteolytic cleavages. As a result, the Notch intracellular domain is released and translocates into the nucleus to engage in transcription regulation [28]. DSL ligands co-expressed in the same cell with Notch receptors are able to inhibit the activation of the receptors (*cis*-inhibition), thereby suppressing the intracellular signal. Recently, the process of *cis*-activation was also described in diverse cell types, extending the range of possible modes of Notch signaling [29]. Both *trans*- and *cis*-interactions are highly sensitive to the relative levels of ligands and receptors, and a switch between two cell states, signal-sending and signal-receiving states, may be generated [30]. Of note, rodent Sertoli cells express Notch receptors and DSL ligands, and thus may be considered as both signal-receiving and signal-sending cells [31,32,33]. Although the expression levels of Notch ligands in Sertoli cells are lower in comparison to germ cells [24], the biological significance of DSL proteins in Sertoli cells has already been reported [32]. Moreover, the presence of Notch receptors in germ cells suggests that Sertoli-cell-derived DSL proteins are implicated in Notch signaling in germ cells, which is important for normal spermatogenesis [31,34,35,36]. The precise control of Notch ligand expression may be, therefore, crucial for the maintenance of seminiferous epithelium homeostasis. Nevertheless, to date, little is known about factors and mechanisms controlling DSL proteins in Sertoli cells. Our previous study revealed that anti-androgen exposure and androgen withdrawal resulted in disturbed expression of DLL1, DLL4, and JAG1 in rat testis in vivo [33], but detailed mechanisms were not determined. Very recently, peroxisome proliferator-activated receptor γ was also identified as a regulator of *Dll4* mRNA expression in boar postnatal testis [37].

In the present study, we aimed to explore the role of testicular sex steroids and their receptors in the regulation of DSL protein expression in rodent Sertoli cells. Primary rat Sertoli cells (PSC) and TM4 Sertoli cell line were treated with either testosterone or 17β-estradiol and pharmacological inhibitors of classical (nuclear) or nonclassical (membrane) androgen and estrogen receptors. In addition, to unveil the precise role of each receptor, the silencing of genes that encode the receptors was performed in TM4 cells.

## 2. Results

### 2.1. The Role of Testosterone in the Control of DLL1, DLL4, and JAG1 in Sertoli Cells

**Figure 1:**
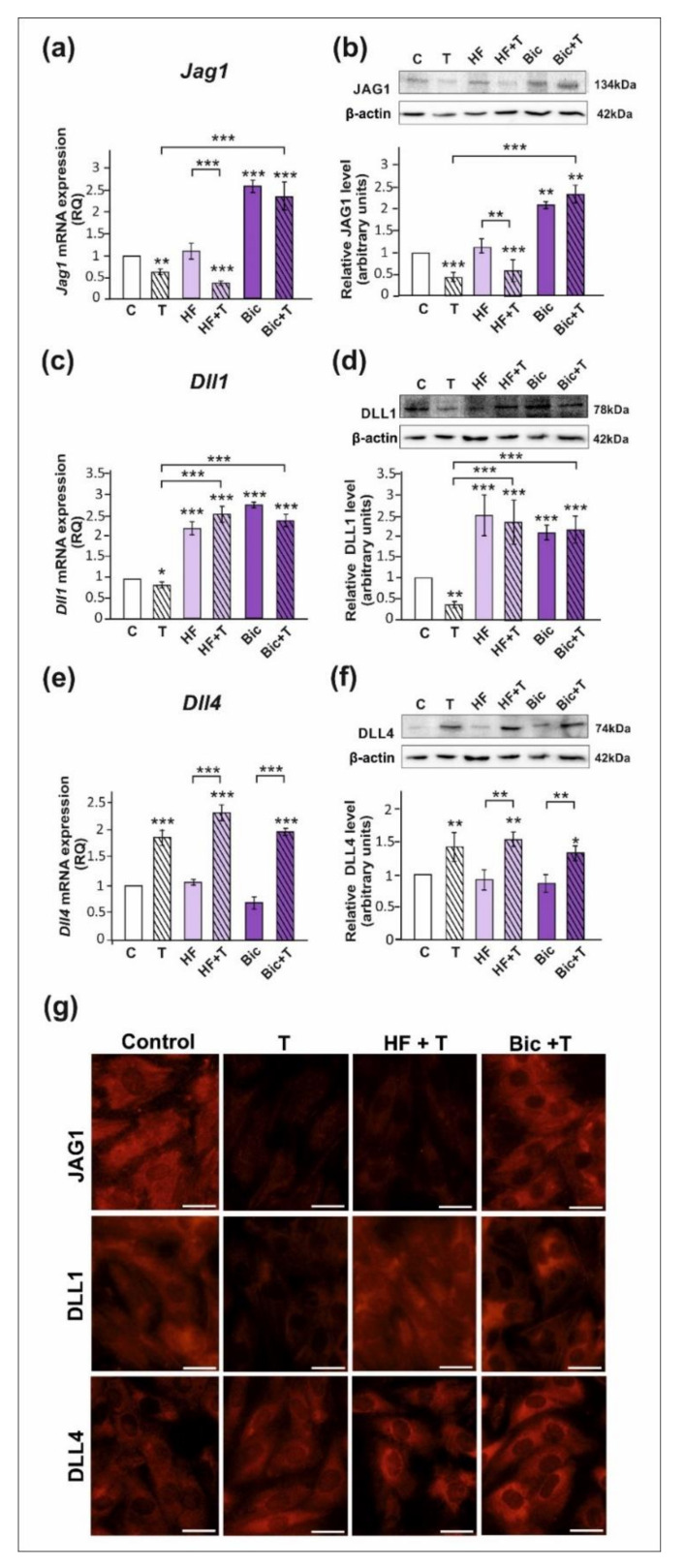
The effect of androgen receptor antagonists on mRNA and protein expression of *Jag1*/JAG1, *Dll1*/DLL1, and *Dll4*/DLL4 in primary rat Sertoli cells. Cells were treated with 10^−8^ M testosterone (T), 10^−4^ M hydroxyflutamide (HF), HF + T, 10^−6^ M bicalutamide (Bic), Bic + T, or vehicle (C) for 24 h. (**a**,**c**,**e**) Relative expression of mRNAs (RQ) was determined using real-time RT-PCR analysis. The expression values of the individual genes were normalized to the mean expression of the reference genes. (**b**,**d**,**f**) Western blot detection of the proteins. The relative level of studied protein was normalized to β-actin. The protein levels within the control group were arbitrarily set at 1. The histograms are the quantitative representation of data (mean ± SD) of three independent experiments, each in triplicate. Significant differences from control values are denoted as * *p* < 0.05, ** *p* < 0.01, and *** *p* < 0.001. (**g**) Immunofluorescence analysis of JAG1, DLL1, and DLL4 expression. Scale bar = 10 µm.

**Figure 2:**
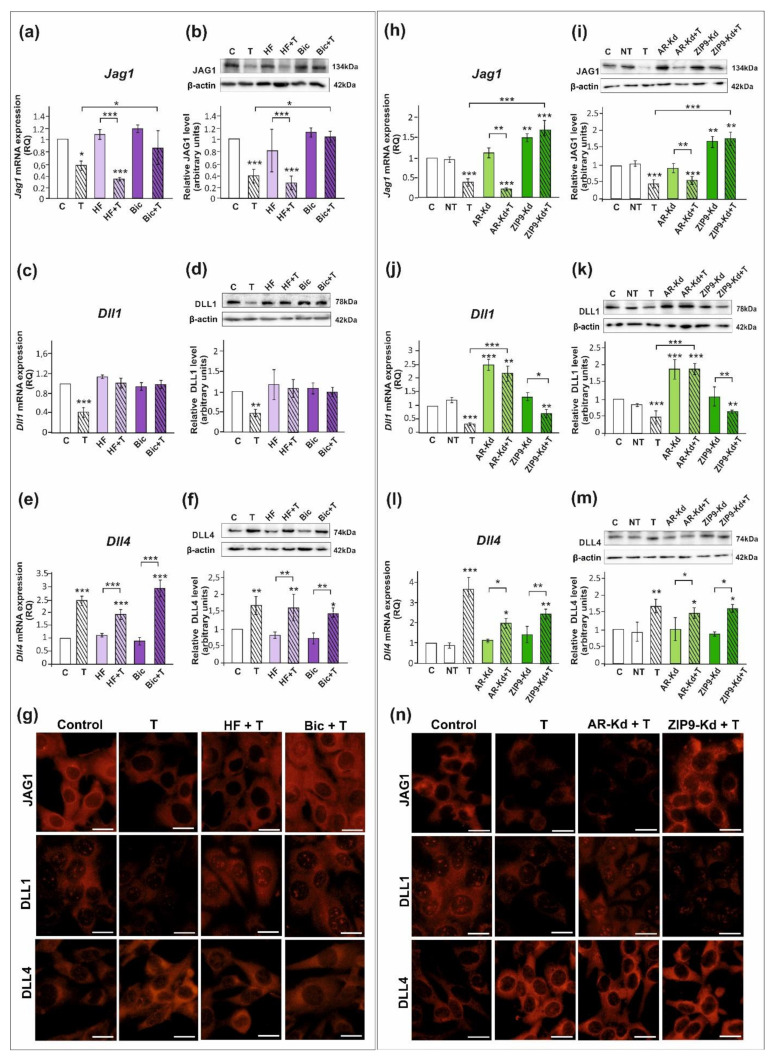
The effect of androgen receptor antagonists or androgen receptor silencing on mRNA and protein expression of *Jag1*/JAG1, *Dll1*/DLL1, and *Dll4*/DLL4 in TM4 Sertoli cell line. (**a**–**g**) Cells were treated with 10^−8^ M testosterone (T), 10^−4^ M hydroxyflutamide (HF), HF + T, 10^−6^ M bicalutamide (Bic), Bic + T, or vehicle (C) for 24 h. (**h**–**n**) Cells were treated with transfection reagent alone (C), transfection reagent + 5 × 10^−8^ M non-targeting siRNA (negative control, NT), transfection reagent + 5 × 10^−8^ M AR siRNA (AR-Kd), or ZIP9 siRNA (ZIP9-Kd). After 24 h, 10^−8^ M T or vehicle was added to the culture. (**a**,**c**,**e**,**h**,**j**,**l**) Relative expression of mRNAs (RQ) was determined using real-time RT-PCR analysis. The expression values of the individual genes were normalized to the mean expression of the reference genes. (**b**,**d**,**f**,**i**,**k**,**m**) Western blot detection of the proteins. The relative level of studied protein was normalized to β-actin. The protein levels within the control group were arbitrarily set at 1. The histograms are the quantitative representation of data (mean ± SD) of three independent experiments, each in triplicate. Significant differences from control values are denoted as * *p* < 0.05, ** *p* < 0.01, and *** *p* < 0.001. (**g**,**n**) Immunofluorescence analysis of JAG1, DLL1, and DLL4 expression. Scale bar = 10 µm.

All studied DSL proteins (DLL1, DLL4, and JAG1), as well as AR and ZIP9, were expressed in both PSC and TM4 Sertoli cells (Figure 1, Figure 2 and Figure S1). As demonstrated by RT-qPCR, Western blot, and immunofluorescence analyses, mRNA and protein expression of JAG1 decreased following testosterone exposure (*p* < 0.01; *p* < 0.001) in both PSC (Figure 1a,b,g) and TM4 cells (Figure 2a,b,g). Exposure to hydroxyflutamide (HF; AR antagonist) had no effect on testosterone-stimulated JAG1 expression, whereas bicalutamide (Bic; AR and ZIP9 antagonist) abrogated the effect of testosterone on JAG1. This suggests the role of membrane androgen receptor ZIP9 in the control of JAG1 expression. To further confirm the mechanisms involved in JAG1 regulation by testosterone, AR and ZIP9 were knocked down in Sertoli cells. Since transfection efficiency in primary Sertoli cells is low ([38]; our unpublished observations), we used TM4 cell line for these experiments. Following knockdown of ZIP9 in TM4 cells, testosterone did not reduce JAG1 expression (*p* < 0.01; *p* < 0.001). In contrast, AR knockdown was ineffective in blocking the action of testosterone (Figure 2h,i). The same effects were also clearly demonstrated using immunofluorescence analysis (Figure 2n). These observations confirmed that mainly ZIP9 is involved in the regulation of JAG1 by testosterone.

Testosterone reduced the expression of mRNA and protein expression of DLL1 (*p* < 0.05; *p* < 0.01), both in PSC (Figure 1c,d) and TM4 cells (Figure 2c,d). Treatment with HF or Bic abolished the effect of testosterone on DLL1 expression. These findings were confirmed by the results of immunofluorescence analysis, showing decreased signal intensity in the cells treated with testosterone alone, but not in the cells incubated with testosterone and HF or Bic, when compared to the control cells (Figure 1g and Figure 2g). Silencing experiments demonstrated that only AR knockdown abolished the effect of testosterone (*p* < 0.001) on DLL1 expression in TM4 cells (Figure 2j,k,n), which corroborates results obtained from experiments with pharmacological inhibitors. These results suggest that the AR is involved in the regulation of DLL1 expression.

In contrast to the effect on DLL1 and JAG1, testosterone enhanced the expression of DLL4 mRNA and protein (*p* < 0.01; *p* < 0.001) in both PSC (Figure 1e,f,g) and TM4 cells (Figure 2e,f,g). Testosterone-induced expression of DLL4 was inhibited neither by HF nor Bic. Likewise, increased DLL4 expression in TM4 cells treated with testosterone (*p* < 0.01; *p* < 0.001) persisted following the AR or ZIP9 knockdown (Figure 2l,m). Immunofluorescence analysis confirmed Western blot data (Figure 2n). Based on these findings DLL4 regulation by testosterone seems to be independent of the AR and ZIP9 activation in Sertoli cells.

### 2.2. The Role of 17β-Estradiol in the Control of DLL1, DLL4, and JAG1 in Sertoli Cells

**Figure 3:**
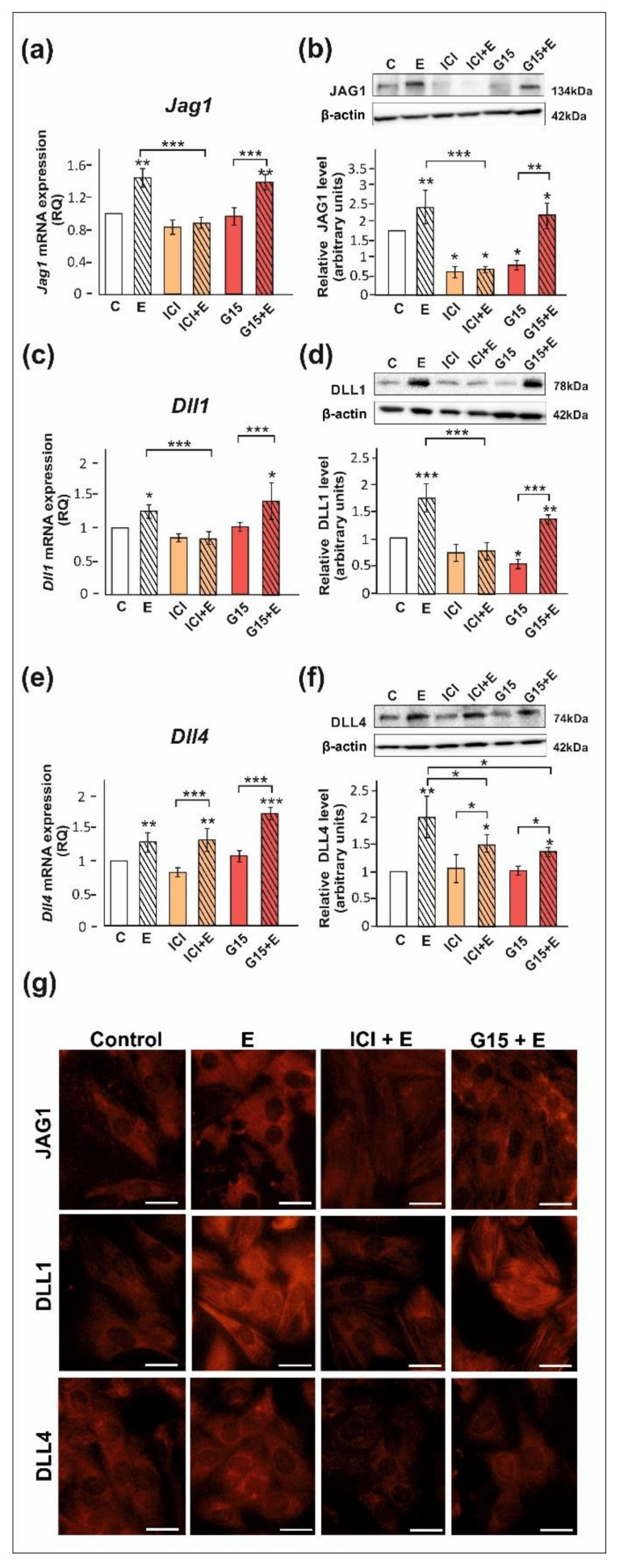
The effect of estrogen receptor antagonists on mRNA and protein expression of *Jag1*/JAG1, *Dll1*/DLL1, and *Dll4*/DLL4 in primary rat Sertoli cells. Cells were treated with 10^−9^ M 17β-estradiol (E), 10^−6^ M ICI 182,780 (ICI), ICI + E, 10^−8^ M G15, G15 + E, or vehicle (C) for 24 h. (**a**,**c**,**e**) Relative expression of mRNAs (RQ) was determined using real-time RT-PCR analysis. The expression values of the individual genes were normalized to the mean expression of the reference genes. (**b**,**d**,**f**) Western blot detection of the proteins. The relative level of studied protein was normalized to β-actin. The protein levels within the control group were arbitrarily set at 1. The histograms are the quantitative representation of data (mean ± SD) of three independent experiments, each in triplicate. Significant differences from control values are denoted as * *p* < 0.05, ** *p* < 0.01, and *** *p* < 0.001. (**g**) Immunofluorescence analysis of JAG1, DLL1, and DLL4 expression. Scale bar = 10 µm.

**Figure 4:**
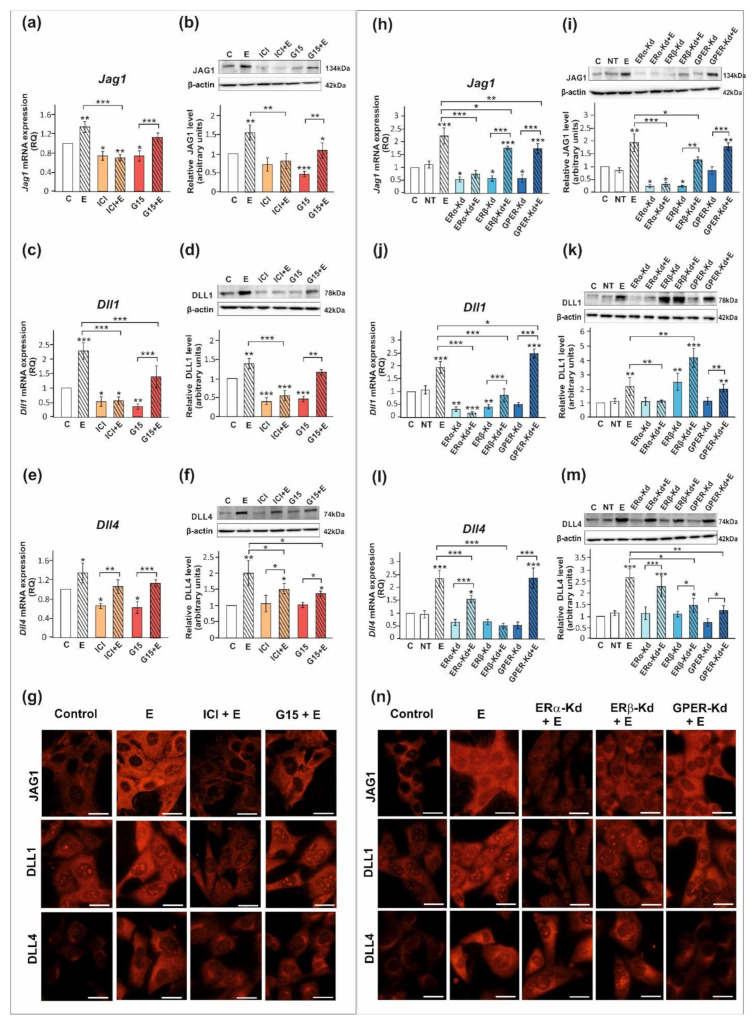
The effect of estrogen receptor antagonists or estrogen receptor silencing on mRNA and protein expression of *Jag1*/JAG1, *Dll1*/DLL1, and *Dll4*/DLL4 in TM4 Sertoli cell line. (**a**–**g**) Cells were treated with 10^−9^ M 17β-estradiol (E), 10^−6^ M ICI 182,780 (ICI), ICI + E, 10^−8^ M G15, G15 + E, or vehicle (C) for 24 h. (**h**–**n**) Cells were treated with transfection reagent alone (C), transfection reagent + 5 × 10^−8^ M non-targeting siRNA (negative control, NT), transfection reagent + 5 × 10^−8^ M ERα siRNA (ERα -Kd), ERβ siRNA (ERβ -Kd), or GPER siRNA (GPER-Kd). After 24 h, 17β-estradiol or vehicle was added to the culture. (**a**,**c**,**e**,**h**,**j**,**l**) Relative expression of mRNAs (RQ) was determined using real-time RT-PCR analysis. The expression values of the individual genes were normalized to the mean expression of the reference genes. (**b**,**d**,**f**,**i**,**k**,**m**) Western blot detection of the proteins. The relative level of studied protein was normalized to β-actin. The protein levels within the control group were arbitrarily set at 1. The histograms are the quantitative representation of data (mean ± SD) of three independent experiments, each in triplicate. Significant differences from control values are denoted as * *p* < 0.05, ** *p* < 0.01, and *** *p* < 0.001. (**g**,**n**) Immunofluorescence analysis of JAG1, DLL1, and DLL4 expression. Scale bar = 10 µm.

We confirmed that ERα, ERβ, and GPER proteins are expressed both in PSC and TM4 cells. The level of ERα protein in PSC is lower than in TM4 cells, whereas protein expression levels of ERβ and GPER are comparable between both cellular models (Figure S1). Exposure of PSC and TM4 to 17β-estradiol resulted in the marked increase in the expression of DLL1, DLL4, and JAG1 mRNA and protein (*p* < 0.05; *p* < 0.01; *p* < 0.001) as detected by RT-qPCR and Western blot, respectively (Figure 3 and Figure 4). In the case of immunofluorescence analysis, a more pronounced effect was found in TM4 cells than in PSC (Figure 3g and Figure 4g,n).

The 17β-estradiol-stimulated increase in JAG1 expression was blocked by ICI 182,780 (ERα/β antagonist) (*p* < 0.01), but not by G15 (GPER antagonist), as detected by RT-qPCR, Western blot, and immunofluorescence analyses (Figure 3a,b,g and Figure 4a,b,g). This indicates that primarily nuclear ERs are involved in the regulation of JAG1 in Sertoli cells. Knockdown experiments demonstrated that ERα silencing completely blocked estradiol-induced upregulation of JAG1 mRNA and protein expression (*p* < 0.001) in TM4 cells. ERβ silencing reduced the effect of estradiol on JAG1 (*p* < 0.05). GPER silencing reduced this effect at mRNA level (*p* < 0.01), but not at the protein level (Figure 4h,i,n). JAG1 regulation by 17β-estradiol in Sertoli cells is, therefore, mediated via the ERα and ERβ, but ERα has a prevailing role.

ICI 182,780 abolished the effect of 17β-estradiol on the expression of DLL1 mRNA and protein (*p* < 0.01; *p* < 0.001), whereas G15 was ineffective in blocking 17β-estradiol action in PSC (Figure 3c,d) and TM4 cells (Figure 4c,d). Immunofluorescence analysis confirmed Western blot data (Figure 3g and Figure 4g). These results suggest a contribution of nuclear ERs to the regulation of DLL1 in Sertoli cells. Knockdown experiments revealed that an increase in *Dll1* mRNA expression in response to 17β-estradiol was blocked by ERα or ERβ silencing (*p* < 0.001), while GPER silencing was inefficacious (Figure 4j). Protein expression of DLL1, however, was suppressed only by ERα silencing (*p* < 0.01) (Figure 4k,n). Thus, in TM4 cells, ERα seems to play a dominant role in the control of DLL1.

Neither ICI 182,780 nor G15 influenced 17β-estradiol-stimulated mRNA expression of *Dll4* in PSC (Figure 3e) and TM4 cells (Figure 4e); however, a decrease in DLL4 protein expression was found when compared to 17β-estradiol-stimulated cells (*p* < 0.05) (Figure 3f and Figure 4f). Moreover, a decreased immunofluorescence signal was observed after exposure of PSC and TM4 cells to ICI 182,780 or G15 (Figure 3g and Figure 4g), which implies the involvement of both nuclear and membrane estrogen receptors. In TM4 cells, estrogen-stimulated mRNA and protein expression of DLL4 was abolished by ERβ knockdown (*p* < 0.05), while ERα silencing had some effect only on *Dll4* mRNA expression (*p* < 0.05). GPER knockdown inhibited the effect of 17β-estradiol on DLL4 protein expression (*p* < 0.01), but not on mRNA expression (Figure 4l,m). Immunofluorescence analysis confirmed Western blot data (Figure 4n). Taken together, ERβ and GPER seem to mediate estrogen action on DLL4 protein expression.

## 3. Discussion

In the present study, we have demonstrated the role of sex steroids and their respective receptors in the control of DSL proteins in rodent Sertoli cells. Our findings provide evidence that androgens and estrogens have an opposite effect on the expression of DLL1 and JAG1 proteins in Sertoli cells: testosterone downregulates their expression, whereas estradiol exerts a stimulatory effect.

Our previous study showed increased expression of JAG1 in the testes of pubertal rats after androgen signaling disruption in vivo [33], suggesting an inhibitory effect of androgens on JAG1. However, in that study, testicular cell types that responded to androgen signaling disruption with changes in JAG1 expression were not identified. The results presented herein revealed that testosterone suppresses JAG1 in Sertoli cells and this effect appeared to be mediated by ZIP9. In agreement, Okada et al. [39] showed that cAMP, a second messenger involved, i.a., in testosterone/ZIP9 signal transduction [10], decreased *Jag1* mRNA level in isolated mouse Sertoli cells. The limited role of the AR in the regulation of JAG1 was also described earlier in DU145 and PC3 prostate cancer cells, in which AR overexpression had almost no effect on the level of JAG1 [40]. JAG1 is known to undergo ectodomain shedding, which is a post-translational event independent of the expression level of mRNA [41], and the ability of Sertoli cells to release JAG1 was documented previously [32]. Martin et al. [42] found that synthetic androgen R1881, an agonist of the AR, increased JAG1 level in prostate cancer cells LNCaP conditioned medium. It cannot, therefore, be excluded that, although *Jag1* gene expression in Sertoli cells is controlled primarily by ZIP9, testosterone action via AR modulates the release of JAG1 ectodomain. This issue remains to be elucidated. In contrast to the effect observed in Sertoli cells, elevated testosterone induced JAG1 expression in the interstitial tissue of murine immature testis, as well as in the activated macrophages [43,44], indicating clearly context-dependent regulation of this protein.

In agreement with the results of our previous in vivo study, which revealed upregulated mRNA and protein expression of DLL1 in the testes of rats in response to flutamide (antiandrogen) or testosterone deprivation [33], herein, it is found that DLL1 expression in Sertoli cells is negatively regulated by testosterone. Based on data from antagonist exposures and knockdown experiments, we demonstrated that this regulation is mediated through the AR, while ZIP9 is not involved. According to our knowledge, these are, to date, the first reports on the role of androgen–AR system in the control of DLL1 expression in mammals. In the only earlier published paper, no effect of testosterone on *Dll1* in the murine gubernaculum was demonstrated [45].

We have recently reported that testosterone increases the activity of the Notch pathway in rat and mouse Sertoli cells, upregulating the expression of Notch1 intracellular domain and the effector genes *Hes1* and *Hey1* [46]. In light of these data, the results of the present study suggest that enhanced Notch pathway activity following testosterone exposure may be, at least to some extent, associated with reduced expression of DLL1 or JAG1 proteins, which potentially prevents *cis*-inhibitory interactions within Sertoli cells. Moreover, our earlier study showed that JAG1 and DLL1 are involved in the control of androgen receptor expression in Sertoli cells. Exposure of the cells to immobilized recombinant JAG1 inhibited ZIP9 mRNA and protein expression, whereas immobilized DLL1 reduced the AR expression [12]. These results, together with our present findings, imply a feedback regulatory mechanism in which androgen receptors are involved in the maintenance of proper expression level of DSL proteins in Sertoli cells.

In contrast to JAG1 and DLL1, DLL4 was positively regulated by testosterone. The stimulatory effect of testosterone on DLL4 in Sertoli cells supports the results of our abovementioned study in which androgen withdrawal in vivo caused decreased expression of DLL4 in pubertal rat testis [33]. Although the previous study also revealed markedly decreased immunostaining for DLL4 in Sertoli cells of flutamide-treated rats, herein, neither the exposure to AR antagonists nor AR knockdown abrogated testosterone-stimulated DLL4 expression. One possible explanation for this discrepancy could be the presence of other AR-expressing cells in the testis of rats used in the former study. It is likely that changes in DLL4 expression in Sertoli cells observed following flutamide treatment in vivo could be mediated by an indirect mechanism (e.g., paracrine), not by AR blockade in Sertoli cells. Notably, specific ablation of AR expression in peritubular myoid cells disturbed Sertoli cell functions and altered gene expression in Sertoli cells [47]. Moreover, in mice lacking testicular AR specifically in the Leydig cells, dysfunction of seminiferous epithelium was reported [48]. Thus, the effect of testosterone on DLL4 expression in Sertoli cells seems to be independent of Sertoli-cell-expressed AR. Furthermore, ZIP9 silencing was also ineffective in preventing the effect of testosterone, indicating the lack of ZIP9 involvement in DLL4 control in Sertoli cells. Further studies are required to elucidate a mechanism of DLL4 regulation by testosterone in Sertoli cells. It cannot be ruled out, however, that this effect is mediated by other receptors, such as G-protein-coupled receptor class C group 6 member A (GPRC6A) or transient receptor potential cation channel subfamily M member 8 (TRPM8), which are localized in rodent Sertoli cells and androgens are among their ligands [49,50,51,52].

The opposite effects of testosterone on different DSL proteins are likely related to the fact that different DSL ligands may trigger different responses of the cell. Recently, Nandagopal et al. [53] proposed the mechanism that leads to either promotion or inhibition of somite myogenesis, depending on the activating ligand, DLL1 or DLL4. Moreover, the results of our previous study demonstrated that different DSL proteins, DLL1 and JAG1, negatively regulate the expression of different androgen-dependent junction proteins in Sertoli cells, claudin 11 or claudin 5, respectively, whereas DLL4 has no effect on their expression [12].

Summing up, androgens exert diverse effects on the expression of DSL proteins in Sertoli cells, which may contribute to the complex regulation of Notch pathway activity in rodent seminiferous epithelium.

Our results demonstrated that 17β-estradiol stimulates the expression of DLL1, DLL4, and JAG1 in both PSC and TM4 cell line. The effect of estradiol on DLL1 and JAG1 expression was clearly abolished by ICI 182, 780, indicating the involvement of nuclear ERs. We found that estradiol upregulated the expression of JAG1 in Sertoli cells acting through both ERα and ERβ, but ERα seems to play the main role in this regulation. Earlier studies on human breast cancer cell line MCF-7 and endometrial stromal cells demonstrated that promoter of JAG1 contains estrogen-responsive elements. Moreover, luciferase reporter analysis revealed that estrogen stimulated the expression of JAG1 via ERα, which is bound to estrogen-responsive element in the JAG1 promoter [54,55]. Interestingly, knockdown of JAG1 in MCF-7 cells resulted in the loss of ERα expression [56], indicating a mutual relationship between these proteins.

In the present study, we showed that 17β-estradiol enhanced the expression of DLL1 acting chiefly through ERα. Increased DLL1 protein expression following estrogen treatment was also found previously in breast cancer cells [57]. The authors reported that ERα knockdown led to a significant decrease in DLL1 protein level but not mRNA level. In contrast, downregulation of both transcript and protein was revealed in TM4 Sertoli cells following ERα silencing, indicating that the loss of ERα may reduce DLL1 level not only by enhancing protein ubiquitination and degradation as reported previously [56], but also by the effect on *Dll1* mRNA expression.

It should be mentioned that the regulation of *Dll1* by estradiol clearly depends on the tissue or cell type. In the uterus, estradiol upregulated *Dll1* mRNA; in the fallopian tube, estrogen acting through ERβ led to the reduction in DLL1 protein, whereas, in human umbilical vein endothelial cells, it had no effect on DLL1 protein expression [58,59,60].

Although the effect of ERβ on estrogen-stimulated expression of JAG1 and DLL1 was less evident in TM4 cells, it cannot be ruled out that, in PSC (that have lower ERα expression), contribution of ERβ in the regulation of these proteins is also important.

Herein, we found estrogen-dependent upregulation of DLL4 in Sertoli cells. Increased mRNA expression of *Dll4* was also detected in human endothelial cells after 17β-estradiol exposure, whereas downregulation of *Dll14* was observed in the vagina and uterus [58,59]. Direct exposure to estrogenic compound bisphenol A enhanced the expression of DLL4 in rat testis explants [61]. Our data provided evidence that both ERβ and GPER are involved in estrogenic stimulation of DLL4 protein in Sertoli cells. The role of GPER in the control of DSL proteins has not been reported yet; however, Pupo et al. [62] demonstrated that estrogen/GPER signaling induced activation of Notch1 and Notch target protein HES1 in breast cancer cells. Of note, GPER silencing in TM4 cells produced significant change in DLL4 expression only at the protein level, which suggests that activity of nonclassical estrogen signaling may be involved in the post-translational regulation of DLL4. Recently, the role of GPER independent of transcriptional activation was reported in the regulation of endothelial glucose transporter 1 [63].

Taken together, although DSL protein expression in Sertoli cells appears to be controlled mainly via nuclear ERs, our findings provided evidence that GPER also contributes to this regulation. Of note, the mechanisms of cross-talk between nuclear and membrane estrogen receptors and their downstream pathways were described [64,65]. Potential involvement of such interactions in the regulation of DSL proteins needs further research.

Based on the available data, the importance of the proper expression levels of DSL proteins in Sertoli cells for the control of germ cell differentiation and survival may be considered. Studies on human testicular samples, which revealed a common expression of Notch2 and Notch4 receptors in seminoma and carcinoma in situ, raised the possibility that Notch signaling plays a role in controlling the mitotic/meiotic switch in primordial germ cells. The authors proposed that dysfunction of this mechanism could result in abnormal chromosomal segregation and the generation of aneuploid cells—precursors for further development to cancer cells [66]. In this context, it cannot be excluded that disruption of other members of the Notch signaling pathway, such as DSL ligands in Sertoli cells, could also lead to abnormal germ cell development and tumor promotion. The significance of proper Notch pathway activity in germ cells was also indicated by Okuda et al. [34], who found that aberrant activation of Notch pathway in spermatogonial stem cells (evoked by a deletion of Nkapl, germ-cell-specific transcriptional suppressor of Notch signaling) caused enhanced germ cell apoptosis and affected several transcriptional factors associated with early germ cell differentiation. In addition, it was demonstrated that exposure of adult rats to the inhibitor of canonical Notch signaling (DAPT) affected the expression of Notch components, including the loss of DLL4 in Sertoli cells at stages VI to IX of seminiferous epithelium cycle. This was accompanied by abnormal morphology of germ cells, a failure of cell division, disturbed spermatid elongation and its premature release, and by increased apoptosis of zygotene spermatocytes and germ cells undergoing the last steps of meiotic division [25]. Notably, meiosis completion, as well as proper spermatid adhesion and release from seminiferous epithelium, are also dependent on androgen action in Sertoli cells [18]. Thus, it may be hypothesized that the control of DSL ligands in Sertoli cells by sex steroids contributes to the effects of these hormones on male germ cell maintenance and the course of spermatogenesis.

Finally, even though DSL proteins are usually described as ligands for Notch receptors, they can also interact with other signaling pathways through their intracellular domains. DSL proteins undergo proteolytic cleavage by gamma-secretase, which generates intracellular domain with nuclear localization [67]. Intracellular domain of DLL1 was shown to mediate transforming growth factor-*β*/activin signaling through binding to Smad2/3 in mouse neural stem cells [68]. In addition, intracellular domain of DLL1 may downregulate Notch receptor activity, disrupting the formation of the Notch intracellular domain and recombination signal binding protein for immunoglobulin kappa J region (RBP-Jk) complex [69]. Recently, JAG1 intracellular domain was identified as a negative regulator of Leydig cell steroidogenesis via Nur77-dependent mechanism [70]. The function of intracellular domains of DSL ligands in Sertoli cells has not been determined to date, but it becomes increasingly apparent that the significance of DSL proteins in these cells may extend well beyond their role as ligands for Notch receptors.

**Figure 5:**
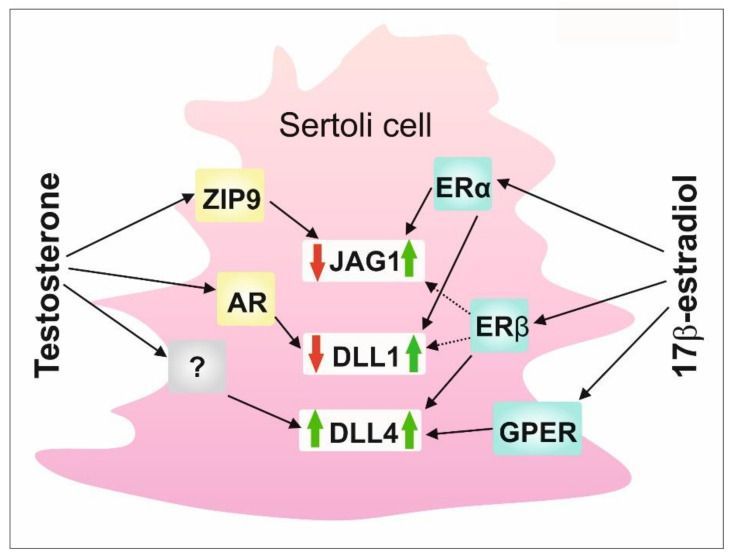
A schematic model of the regulation of DSL proteins by sex steroids and their receptors in rodent Sertoli cells. AR—nuclear androgen receptor; DLL1—Delta-like 1; DLL4—Delta-like 4; ERα—estrogen receptor α; ERβ—estrogen receptor β; GPER - G protein-coupled estrogen receptor; JAG1—Jagged1; ZIP9—ZRT-and Irt-like protein 9.

To summarize, data presented herein highlight a substantial role of the co-operation of classical and nonclassical signaling pathways triggered by androgens and estrogens in maintaining the proper expression of DSL proteins in Sertoli cells (Figure 5). Thus, a delicate balance between testicular androgens and estrogens and their nuclear and membrane receptors existing in seminiferous epithelium seems to be important for appropriate Notch signaling activity in the testis, and, thereby, for seminiferous epithelium homeostasis and spermatogenesis.

## 4. Materials and Methods

### 4.1. Cell Cultures and Treatments

Primary cultures of Sertoli cells (PSC) were isolated from the testes of 20-day-old rats using enzymatic digestion and washing, according to previously published protocol [71,72]. Briefly, PSC were seeded at 0.5 × 10^6^ cells/cm^2^ in 6-well plates or on coverslips and incubated in Dulbecco’s modified Eagle medium (DMEM) supplemented with growth factors and an antibiotic in a humidified atmosphere of 95% air and 5% CO_2_ (*vol*/*vol*) at 35 °C. At 48 h after plating, cultures were treated with a hypotonic buffer (20 mM Tris pH 7.4 at 22 °C) to lyse contaminating germ cells and obtain PSC with 98% purity. Cells were treated with: (i) 10^−8^ M testosterone (Cat NO. 86500; Sigma-Aldrich, St. Louis, MO, USA), anti-androgens 10^−4^ M hydroxyflutamide (HF; Cat NO. H4166; Sigma-Aldrich, St. Louis, MO, USA), or 10^−6^ M bicalutamide (Bic) (Cat NO. B9061; Sigma-Aldrich, St. Louis, MO, USA), alone or with the addition of 10^−8^ M testosterone for 24 h; (ii) 10^−9^ M 17β-estradiol (Cat NO. E2758; Sigma-Aldrich, St. Louis, MO, USA), 10^−6^ M ERα/β antagonist ICI 182,780 (ICI, Cat NO. 5.31042; Sigma-Aldrich, St. Louis, MO, USA), or 10^−8^ M selective GPER antagonist G15 (Cat NO. 3678, Tocris Bioscience, Bristol, UK), alone or with the addition of 10^−9^ M 17β-estradiol. All compounds were dissolved in dimethyl sulfoxide (DMSO) before addition to the culture medium to obtain the above final concentrations. Control cells were incubated in the presence of the vehicle only (0.01% DMSO).

Murine Sertoli cell line TM4 (Cat NO. CRL-1715; ATCC, Manassas, VA, USA) was cultured in DMEM supplemented with 10% fetal bovine serum (FBS; Thermo Fisher Scientific, Rocheford, IL, USA) at 37 °C in 5% CO_2_. Properties of TM4 cell line were evaluated as described previously [12]. Before the experiments, cells were serum starved for 24 h. The same cell concentration (0.5 × 10^6^/cm^2^) was used in all experimental groups. In the first experiment, the cells cultured in plates or on the coverslips were treated with steroid hormones or steroid hormone receptor antagonists according to the protocol described above for PSC. In the second experiment, TM4 cells were seeded at 0.1 × 10^5^ cells/cm^2^ in 6-well plates or on coverslips and transfected with Silencer Select siRNAs (AR-specific siRNA assay ID: s62547; ZIP9-specific siRNA assay ID: s116149; ERα-specific siRNA assay ID: s65686; ERβ-specific siRNA assay ID: s65689; GPER-specific siRNA assay ID: s94713, Thermo Fisher Scientific, Rocheford, IL, USA) using Lipofectamine RNAiMAX Transfection Reagent (Thermo Fisher Scientific, Rocheford, IL, USA) in serum-free Opti-MEM (Cat NO. 11058021; Life Technologies, Gaithersburg, MD, USA) according to the manufacturer’s instructions. Negative control cells were treated with transfection reagent alone or transfection reagent plus Silencer Select Negative Control No. 1 (nontargeting siRNA; Cat NO. 4404020; Thermo Fisher Scientific, Rocheford, IL, USA). Silencer Select GAPDH Positive Control siRNA (Cat NO. 4390849; Thermo Fisher Scientific, Rocheford, IL, USA) was used for positive control. Transfection efficiencies were determined with Western blot analysis based on the relative expression levels of the receptor proteins in transfected cell populations vs. control cultures. Transfection efficiencies were: 87 ± 2% for AR siRNA, 73 ± 5% for ZIP9 siRNA, 76% ± 9% for ERα siRNA, 80% ± 1% for ERβ siRNA, and 68% ± 9% for GPER siRNA. After 24 h, cells were washed to remove silencing duplexes and transfection medium. Cells were treated with testosterone, 17β-estradiol, or a vehicle for 24h as described above.

### 4.2. RNA Isolation, Reverse Transcription, and Quantitative RT-PCR (RT-qPCR)

Total RNA was extracted with TRIzol^®^ reagent (Cat NO. 15596026; Life Technologies, Gaithersburg, MD, USA) according to the manufacturer’s instructions. Residual DNA was removed with TURBO DNA-free Kit (Cat NO. AM1907; Ambion, Austin, TX, USA). The yield and quality of the RNA were evaluated by checking the A260:A280 ratio (NanoDrop ND2000 Spectrophotometer, Thermo Scientific, Rocheford, IL, USA) and by electrophoresis. High-Capacity cDNA Reverse Transcription Kit (Cat NO. 4368814; Applied Biosystems, Carlsbad, CA, USA) was used to generate cDNA. For each RNA sample, reactions in the absence of RT were run to appraise genomic DNA contamination. RT-qPCR analyses were performed with the 10 ng cDNA templates, 0.5 μM primers (Institute of Biochemistry and Biophysics, Polish Academy of Sciences, Table 1), and SYBR Green master mix (Cat NO. 4309155; Applied Biosystems, Carlsbad, CA, USA) in a final volume of 10 μL with the StepOne Real-time PCR system (Applied Biosystems, Carlsbad, CA, USA). PCR conditions: 55 °C for 2 min, 94 °C for 10 min, followed by denaturation temperature 95 °C for 15 s and annealing temperature for 60 s to determine the cycle threshold (Ct) for quantitative measurement. Amplification efficiency was between 97% and 104%. Melting curve analysis and agarose gel electrophoresis were used to confirm amplification specificity. Negative control reactions corresponding to RT reaction without the reverse transcriptase enzyme and a blank sample were carried out. The reference gene candidates were tested on experimental and control samples. The Microsoft Excel-based application NormFinder was used to analyze the expression stability of commonly used reference genes. Based on these analyses, housekeeping genes for normalizing RNA expression were selected: *Rn18s*, *B2m*, *Actb*, *Rpl13a*, *Hprt1*, and *Gapdh.* mRNA expressions were normalized to the mean expression of the reference genes (relative quantification, RQ = 1) with the use of the 2^−ΔΔCt^ method [73].

### 4.3. Western Blot Analysis

Lysates were obtained by cell sonification with Tris/EDTA buffer (50 mM Tris, 1 mM EDTA, pH 7.5) containing protease inhibitors (Cat NO. P8340; Sigma-Aldrich, St. Louis, MO, USA). Protein concentration was determined with DC protein assay kit (Bio-Rad Laboratories, Hercules, CA, USA) using BSA as a standard. Proteins were resolved by SDS-PAGE under reducing conditions, transferred to polyvinylidene difluoride membranes (Sigma-Aldrich, St. Louis, MO, USA), and detected by immunoblotting as previously reported in detail [60]. Primary antibodies used in the analyses were: anti-JAG1 (1:3000; Cat NO. PA5–72843, Thermo Fisher Scientific, Rocheford, IL, USA), anti-DLL1 (1:1000; Cat NO. SAB2100593, Sigma-Aldrich, St. Louis, MO, USA), and anti-DLL4 (1:2000; Cat NO. AB7280, Abcam, Cambrige, UK). Secondary horseradish peroxidase-conjugated antibody (1:3000; Cat NO. 31460, Thermo Fisher Scientific, Rocheford, IL, USA), followed by enhanced chemiluminescence, was used to detect targeted protein bands. Bands were visualized with a ChemiDocTM XRS+ System (Bio–Rad Labs., München, Germany). The molecular weights of targeted proteins were assessed by reference to standard proteins (PageRuler Prestained Protein Ladder, Thermo Fisher Scientific, Rocheford, IL, USA). All immunoblots were stripped (25 mM glycine-HCl, 1% (*w*/*v*) sodium dodecyl sulfate, pH 2.1 for 30 min) and reprobed with an antibody against β-actin (1:3000; Cat NO. A2228, Sigma-Aldrich, St. Louis, MO, USA), which served as the protein loading control, followed by secondary horseradish peroxidase-conjugated antibody (1:3000, Cat NO. 1706516, Bio-Rad Labs., München, Germany). Relative intensities of protein bands were quantified by the ImageLab software (Bio-Rad Labs., München, Germany).

### 4.4. Immunofluorescence

Immunofluorescence was performed on PSC and TM4 cells seeded on coverslips. The cells were washed with phosphate-buffered saline (PBS), fixed with cold methanol–acetone, and immunostained as described previously [74]. Analysis was performed with the corresponding primary antibodies listed in Section 4.3. (anti-JAG1, dilution 1:100; anti-DLL1, dilution 1:50; anti-DLL4, dilution 1:50) and Cy3-conjugated goat anti-Rabbit IgG (1:200; Cat NO. A10520; Thermo Fischer Scientific, Rocheford, IL, USA) secondary antibody. Images were captured with epifluorescence microscope Nikon Eclipse Ni (Nikon Instech Co., Tokyo, Japan). No fluorescence was observed in the negative controls, where the respective primary antibodies were omitted (not shown).

### 4.5. Statistical Analysis

Each data point was a mean ± SD of the results from three independent experiments. Normality and the homogeneity of variance were tested with Shapiro–Wilk W-test and Levene’s test, respectively. Statistical significance was assessed using one-way ANOVA, followed by Tukey’s post hoc comparison test. Statistical analyses were performed on raw data using Statistica 10 software (StatSoft Inc., Tulsa, OK, USA). Data were considered statistically significant at * *p* < 0.05, ** *p* < 0.01, *** *p* < 0.001.

## Figures and Tables

**Table 1:** Sequences of forward and reverse primers.

Gene	Forward Sequence	Reverse Sequence	Product Size (bp)	Annealing Temp (°C)
**Mouse**				
*Actb*	AAGAGCTATGAGCTGCCTGA	TACGGATGTCAACGTCACAC	160	58
*B2m*	GGCCTGTATGCTATCCAGAA	GAAAGACCAGTCCTTGCTGA	198	58
*Dll1*	TCAGATAACCCTGACGGAGGC	AGGTAAGAGTTGCCGAGGTCC	185	56
*Dll4*	GCTGGAAGTGGATTGTGG	CTTGTCGCTGTGAGGATAC	405	51
*Gapdh*	CTGGAGAAACCTGCCAAGTA	TGTTGCTGTAGCCGTATTCA	223	58
*Hprt1*	GCTGACCTGCTGGATTACAT	TTGGGGCTGTACTGCTTAAC	242	58
*Jag1*	AACTGGTACCGGTGCGAA	TGATGCAAGATCTCCCTGAAAC	216	54
*Rn18s*	CTCTGGTTGCTCTGTGCAGT	GGCTCCTTGTAGGGGTTCTC	455	52
*Rpl13a*	ATGACAAGAAAAAGCGGATG	CTTTTCTGCCTGTTTCCGTA	215	58
**Rat**				
*Actb*	CACACTGTGCCCATCTATGA	CCGATAGTGATGACCTGACG	272	58
*B2m*	TGCTACGTGTCTCAGTTCCA	GCTCCTTCAGAGTGACGTGT	196	58
*Dll1*	TCAGATAACCCTGACGGAGGC	AGGTAAGAGTTGCCGAGGTCC	185	56
*Dll4*	GCTGGAAGTGGATTGTGG	CTTGTCGCTGTGAGGATAC	405	51
*Gapdh*	AGACAGCCGCATCTTCTTGT	CTTGCCGTGGGTAGAGTCAT	207	58
*Hprt1*	GACTTTGCTTTCCTTGGTCA	AGTCAAGGGCATATCCAACA	152	58
*Jag1*	AACTGGTACCGGTGCGAA	TGATGCAAGATCTCCCTGAAAC	216	54
*Rn18s*	GCCGCGGTAATTCCAGCTCCA	CCCGCCCGCTCCCAAGATC	320	61
*Rpl13a*	GTGAGGGCATCAACATTTCT	CATCCGCTTTTTCTTGTCAT	242	58

## Data Availability

The data presented in this study are available on request from the corresponding author.

## Supplementary Materials

The following supporting information can be downloaded at: https://www.mdpi.com/article/10.3390/ijms23042284/s1.

## References

[1] McKinnell C., Atanassova N., Williams K., Fisher J.S., Walker M., Turner K.J., Saunders T.K., Sharpe R.M. (2001). Suppression of androgen action and the induction of gross abnormalities of the reproductive tract in male rats treated neonatally with diethylstilbestrol. J. Androl..

[2] Rivas A., Fisher J.S., McKinnell C., Atanassova N., Sharpe R.M. (2002). Induction of reproductive tract developmental abnormalities in the male rat by lowering androgen production or action in combination with a low dose of diethylstilbestrol: Evidence for importance of the androgen-estrogen balance. Endocrinology.

[3] Rivas A., McKinnell C., Fisher J.S., Atanassova N., Williams K., Sharpe R.M. (2003). Neonatal coadministration of testosterone with diethylstilbestrol prevents diethylstilbestrol induction of most reproductive tract abnormalities in male rats. J. Androl..

[4] Li X., Rahman N. (2008). Impact of androgen/estrogen ratio: Lessons learned from the aromatase over-expression mice. Gen. Comp. Endocrinol..

[5] Pardyak L., Kaminska A., Brzoskwinia M., Hejmej A., Kotula-Balak M., Jankowski J., Ciereszko A., Bilinska B. (2018). Differences in aromatase expression and steroid hormone concentrations in the reproductive tissues of male domestic turkeys (Meleagris gallopavo) with white and yellow semen. Br. Poult. Sci..

[6] Misiakiewicz-Has K., Pilutin A., Wiszniewska B. (2021). Influence of hormonal imbalance on the integrity of seminiferous epithelium in the testes of adult rats chronically exposed to letrozole and rats exposed to soya isoflavones during the prenatal period, lactation, and up to sexual maturity. Reprod. Biol..

[7] Cooke P.S., Nanjappa M.K., Ko C., Prins G.S., Hess R.A. (2017). Estrogens in male physiology. Physiol. Rev..

[8] Hess R.A., Cooke P.S. (2018). Estrogen in the male: A historical perspective. Biol. Reprod..

[9] Lucas T.F., Royer C., Siu E.R., Lazari M.F., Porto C.S. (2010). Expression and signaling of G protein-coupled estrogen receptor 1 (GPER) in rat Sertoli cells. Biol. Reprod..

[10] Berg A.H., Rice C.D., Rahman M.S., Dong J., Thomas P. (2014). Identification and characterization of membrane androgen receptors in the ZIP9 zinc transporter subfamily: I. Discovery in female atlantic croaker and evidence ZIP9 mediates testosterone-induced apoptosis of ovarian follicle cells. Endocrinology.

[11] Bulldan A., Dietze R., Shihan M., Scheiner-Bobis G. (2016). Non-classical testosterone signaling mediated through ZIP9 stimulates claudin expression and tight junction formation in Sertoli cells. Cell. Signal..

[12] Kamińska A., Pardyak L., Marek S., Wróbel K., Kotula-Balak M., Bilińska B., Hejmej A. (2020). Notch signaling regulates nuclear androgen receptor AR and membrane androgen receptor ZIP9 in mouse Sertoli cells. Andrology.

[13] Duliban M., Gurgul A., Szmatola T., Pawlicki P., Milon A., Arent Z.J., Grzmil P., Kotula-Balak M., Bilinska B. (2020). Mouse testicular transcriptome after modulation of non-canonical oestrogen receptor activity. Reprod. Fertil. Dev..

[14] Pavlovich C.P., King P., Goldstein M., Schlegel P.N. (2001). Evidence of a treatable endocrinopathy in infertile men. J. Urol..

[15] Hejmej A., Bilińska B. (2008). The effects of cryptorchidism on the regulation of steroidogenesis and gap junctional communication in equine testes. Endokrynol. Pol..

[16] Lardone M.C., Argandoña F., Flórez M., Parada-Bustamante A., Ebensperger M., Palma C., Piottante A., Castro A. (2017). Overexpression of CYP19A1 aromatase in Leydig cells is associated with steroidogenic dysfunction in subjects with Sertoli cell-only syndrome. Andrology.

[17] Kaufman J.M., Vermeulen A. (2005). The decline of androgen levels in elderly men and its clinical and therapeutic implications. Endocr. Rev..

[18] O’Hara L., Smith L.B. (2015). Androgen receptor roles in spermatogenesis and infertility. Best Pract. Res. Clin. Endocrinol. Metab..

[19] Lucas T.F., Pimenta M.T., Pisolato R., Lazari M.F., Porto C.S. (2011). 17β-estradiol signaling and regulation of Sertoli cell function. Spermatogenesis.

[20] Martins A.D., Alves M.G., Simões V.L., Dias T.R., Rato L., Moreira P.I., Socorro S., Cavaco J.E., Oliveira P.F. (2013). Control of Sertoli cell metabolism by sex steroid hormones is mediated through modulation in glycolysis-related transporters and enzymes. Cell Tissue Res..

[21] Chimento A., De Luca A., Nocito M.C., Avena P., La Padula D., Zavaglia L., Pezzi V. (2020). Role of GPER-Mediated Signaling in Testicular Functions and Tumorigenesis. Cells.

[22] Macheroni C., Lucas T., Porto C.S. (2020). The role of estrogen receptors in rat Sertoli cells at different stages of development. Heliyon.

[23] Huang Z., Rivas B., Agoulnik A.I. (2013). NOTCH1 gain of function in germ cells causes failure of spermatogenesis in male mice. PLoS ONE.

[24] Garcia T.X., Farmaha J.K., Kow S., Hofmann M.C. (2014). RBPJ in mouse Sertoli cells is required for proper regulation of the testis stem cell niche. Development.

[25] Murta D., Batista M., Trindade A., Silva E., Henrique D., Duarte A., Lopes-da-Costa L. (2014). In vivo notch signaling blockade induces abnormal spermatogenesis in the mouse. PLoS ONE.

[26] Parekh P.A., Garcia T.X., Waheeb R., Jain V., Gandhi P., Meistrich M.L., Shetty G., Hofmann M.C. (2019). Undifferentiated spermatogonia regulate *Cyp26b1* expression through NOTCH signaling and drive germ cell differentiation. FASEB J..

[27] Kopan R., Ilagan M.X. (2009). The canonical Notch signaling pathway: Unfolding the activation mechanism. Cell.

[28] Nichols J.T., Miyamoto A., Weinmaster G. (2007). Notch signaling--constantly on the move. Traffic.

[29] Nandagopal N., Santat L.A., Elowitz M.B. (2019). *Cis-*activation in the Notch signaling pathway. Elife.

[30] Sprinzak D., Lakhanpal A., Lebon L., Santat L.A., Fontes M.E., Anderson G.A., Garcia-Ojalvo J., Elowitz M.B. (2010). Cis-interactions between Notch and Delta generate mutually exclusive signaling states. Nature.

[31] Murta D., Batista M., Silva E., Trindade A., Henrique D., Duarte A., Lopes-da-Costa L. (2013). Dynamics of Notch pathway expression during mouse testis post-natal development and along the spermatogenic cycle. PLoS ONE.

[32] Campese A.F., Grazioli P., de Cesaris P., Riccioli A., Bellavia D., Pelullo M., Padula F., Noce C., Verkhovskaia S., Filippini A. (2014). Mouse Sertoli cells sustain de novo generation of regulatory T cells by triggering the notch pathway through soluble JAGGED1. Biol. Reprod..

[33] Kamińska A., Marek S., Pardyak L., Brzoskwinia M., Pawlicki P., Bilińska B., Hejmej A. (2020). Disruption of androgen signaling during puberty affects Notch pathway in rat seminiferous epithelium. Reprod. Biol. Endocrinol..

[34] Okuda H., Kiuchi H., Takao T., Miyagawa Y., Tsujimura A., Nonomura N., Miyata H., Okabe M., Ikawa M., Kawakami Y. (2015). A novel transcriptional factor Nkapl is a germ cell-specific suppressor of Notch signaling and is indispensable for spermatogenesis. PLoS ONE.

[35] Okada R., Fujimagari M., Koya E., Hirose Y., Sato T., Nishina Y. (2017). Expression Profile of NOTCH3 in Mouse Spermatogonia. Cells Tissues Organs.

[36] Lustofin S., Kamińska A., Pardyak L., Pawlicki P., Brzoskwinia M., Szpręgiel I., Bilińska B., Hejmej A. (2022). Follicle-stimulating hormone regulates Notch signalling in seminiferous epithelium of continuously and seasonally breeding rodents. Reprod. Fertil. Dev..

[37] Duliban M., Pawlicki P., Gurgul A., Tuz R., Arent Z., Kotula-Balak M., Tarasiuk K. (2021). Proliferator-activated receptor γ, but not α or g-protein coupled estrogen receptor drives functioning of postnatal boar testis-next generation sequencing analysis. Animals.

[38] Garcia T.X., Parekh P., Gandhi P., Sinha K., Hofmann M.C. (2017). The NOTCH Ligand JAG1 Regulates GDNF Expression in Sertoli Cells. Stem. Cells Dev..

[39] Okada R., Hara T., Sato T., Kojima N., Nishina Y. (2016). The mechanism and control of Jagged1 expression in Sertoli cells. Regen. Ther..

[40] Yu Y., Zhang Y., Guan W., Huang T., Kang J., Sheng X., Qi J. (2014). Androgen receptor promotes the oncogenic function of overexpressed Jagged1 in prostate cancer by enhancing cyclin B1 expression via Akt phosphorylation. Mol. Cancer Res..

[41] Guo L., Eisenman J.R., Mahimkar R.M., Peschon J.J., Paxton R.J., Black R.A., Johnson R.S. (2002). A proteomic approach for the identification of cell-surface proteins shed by metalloproteases. Mol. Cell. Proteom..

[42] Martin D.B., Gifford D.R., Wright M.E., Keller A., Yi E., Goodlett D.R., Aebersold R., Nelson P.S. (2004). Quantitative proteomic analysis of proteins released by neoplastic prostate epithelium. Cancer Res..

[43] Guo D., Zhang H., Liu L., Wang L., Cheng Y., Qiao Z. (2004). Testosterone influenced the expression of Notch1, Notch2 and Jagged1 induced by lipopolysaccharide in macrophages. Exp. Toxicol. Pathol..

[44] Defalco T., Saraswathula A., Briot A., Iruela-Arispe M.L., Capel B. (2013). Testosterone levels influence mouse fetal Leydig cell progenitors through notch signaling. Biol. Reprod..

[45] Yuan F.P., Lin D.X., Rao C.V., Lei Z.M. (2006). Cryptorchidism in LhrKO animals and the effect of testosterone-replacement therapy. Hum. Reprod..

[46] Kamińska A., Marek S., Pardyak L., Brzoskwinia M., Bilinska B., Hejmej A. (2020). Crosstalk between androgen-ZIP9 signaling and Notch pathway in rodent Sertoli cells. Int. J. Mol. Sci..

[47] Welsh M., Saunders P.T., Atanassova N., Sharpe R.M., Smith L.B. (2009). Androgen action via testicular peritubular myoid cells is essential for male fertility. FASEB J..

[48] O’Hara L., McInnes K., Simitsidellis I., Morgan S., Atanassova N., Slowikowska-Hilczer J., Kula K., Szarras-Czapnik M., Milne L., Mitchell R.T. (2015). Autocrine androgen action is essential for Leydig cell maturation and function, and protects against late-onset Leydig cell apoptosis in both mice and men. FASEB J..

[49] Pi M., Chen L., Huang M.Z., Zhu W., Ringhofer B., Luo J., Christenson L., Li B., Zhang J., Jackson P.D. (2008). GPRC6A null mice exhibit osteopenia, feminization and metabolic syndrome. PLoS ONE.

[50] Asuthkar S., Velpula K.K., Elustondo P.A., Demirkhanyan L., Zakharian E. (2015). TRPM8 channel as a novel molecular target in androgen-regulated prostate cancer cells. Oncotarget.

[51] Borowiec A.S., Sion B., Chalmel F., Rolland A.D., Lemonnier L., De Clerck T., Bokhobza A., Derouiche S., Dewailly E., Slomianny C. (2016). Cold/menthol TRPM8 receptors initiate the cold-shock response and protect germ cells from cold-shock-induced oxidation. FASEB J..

[52] Ye R., Pi M., Nooh M.M., Bahout S.W., Quarles L.D. (2019). Human GPRC6A mediates testosterone-induced mitogen-activated protein kinases and mTORC1 signaling in prostate cancer cells. Mol. Pharmacol..

[53] Nandagopal N., Santat L.A., LeBon L., Sprinzak D., Bronner M.E., Elowitz M.B. (2018). Dynamic ligand discrimination in the Notch signaling pathway. Cell.

[54] Soares R., Balogh G., Guo S., Gärtner F., Russo J., Schmitt F. (2004). Evidence for the Notch signaling pathway on the role of estrogen in angiogenesis. Mol. Endocrinol..

[55] Li N., Zhang L., Li Q., Du Y., Liu H., Liu Y., Xiong W. (2018). Notch activity mediates oestrogen-induced stromal cell invasion in endometriosis. Reproduction.

[56] Buckley N.E., Nic An tSaoir C.B., Blayney J.K., Oram L.C., Crawford N.T., D’Costa Z.C., Quinn J.E., Kennedy R.D., Harkin D.P., Mullan P.B. (2013). BRCA1 is a key regulator of breast differentiation through activation of Notch signaling with implications for anti-endocrine treatment of breast cancers. Nucleic Acids Res..

[57] Kumar S., Srivastav R.K., Wilkes D.W., Ross T., Kim S., Kowalski J., Chatla S., Zhang Q., Nayak A., Guha M. (2019). Estrogen-dependent DLL1-mediated Notch signaling promotes luminal breast cancer. Oncogene.

[58] Nakamura T., Miyagawa S., Katsu Y., Sato T., Iguchi T., Ohta Y. (2012). Sequential changes in the expression of Wnt- and Notch-related genes in the vagina and uterus of ovariectomized mice after estrogen exposure. In Vivo.

[59] Caliceti C., Aquila G., Pannella M., Morelli M.B., Fortini C., Pinton P., Bonora M., Hrelia S., Pannuti A., Miele L. (2013). 17β-estradiol enhances signaling mediated by VEGF-A-delta-like ligand 4-notch1 axis in human endothelial cells. PLoS ONE.

[60] Zhu M., Iwano T., Takeda S. (2019). Estrogen and EGFR pathways regulate Notch signaling in opposing directions for multi-ciliogenesis in the fallopian tube. Cells.

[61] Kamińska A., Pardyak L., Marek S., Górowska-Wójtowicz E., Kotula-Balak M., Bilińska B., Hejmej A. (2018). Bisphenol A and dibutyl phthalate affect the expression of juxtacrine signaling factors in rat testis. Chemosphere.

[62] Pupo M., Pisano A., Abonante S., Maggiolini M., Musti A.M. (2014). GPER activates Notch signaling in breast cancer cells and cancer-associated fibroblasts (CAFs). Int. J. Biochem. Cell Biol..

[63] Boscaro C., Carotti M., Albiero M., Trenti A., Fadini G.P., Trevisi L., Sandonà D., Cignarella A., Bolego C. (2020). Non-genomic mechanisms in the estrogen regulation of glycolytic protein levels in endothelial cells. FASEB J..

[64] Björnström L., Sjöberg M. (2005). Mechanisms of estrogen receptor signaling: Convergence of genomic and nongenomic actions on target genes. Mol. Endocrinol..

[65] Silva E., Kabil A., Kortenkamp A. (2010). Cross-talk between non-genomic and genomic signaling pathways—Distinct effect profiles of environmental estrogens. Toxicol. Appl. Pharmacol..

[66] Adamah D.J., Gokhale P.J., Eastwood D.J., Rajpert De-Meyts E., Goepel J., Walsh J.R., Moore H.D., Andrews P.W. (2006). Dysfunction of the mitotic:meiotic switch as a potential cause of neoplastic conversion of primordial germ cells. Int. J. Androl..

[67] LaVoie M.J., Selkoe D.J. (2003). The Notch ligands, Jagged and Delta, are sequentially processed by alpha-secretase and presenilin/gamma-secretase and release signaling fragments. J. Biol. Chem..

[68] Hiratochi M., Nagase H., Kuramochi Y., Koh C.S., Ohkawara T., Nakayama K. (2007). The Delta intracellular domain mediates TGF-beta/Activin signaling through binding to Smads and has an important bi-directional function in the Notch-Delta signaling pathway. Nucleic Acids Res..

[69] Jung J., Mo J.S., Kim M.Y., Ann E.J., Yoon J.H., Park H.S. (2011). Regulation of Notch1 signaling by Delta-like ligand 1 intracellular domain through physical interaction. Mol. Cells.

[70] Kumar S., Park H.S., Lee K. (2020). Jagged1 intracellular domain modulates steroidogenesis in testicular Leydig cells. PLoS ONE.

[71] Mruk D.D., Cheng C.Y. (2011). An in vitro system to study Sertoli cell blood-testis barrier dynamics. Methods Mol. Biol..

[72] Chojnacka K., Zarzycka M., Hejmej A., Mruk D.D., Gorowska E., Kotula-Balak M., Klimek M., Bilinska B. (2016). Hydroxyflutamide affects connexin 43 via the activation of PI3K/Akt-dependent pathway but has no effect on the crosstalk between PI3K/Akt and ERK1/2 pathways at the Raf-1 kinase level in primary rat Sertoli cells. Toxicol. In Vitro.

[73] Livak K.J., Schmittgen T.D. (2001). Analysis of relative gene expression data using real-time quantitative PCR and the 2(-Delta Delta C(T)) Method. Methods.

[74] Bilinska B., Hejmej A., Kotula-Balak M. (2018). Preparation of testicular samples for histology and immunohistochemistry. Methods Mol. Biol..

