# Supplementary material for: Nuclear and Membrane Receptors for Sex Steroids Are Involved in the Regulation of Delta/Serrate/LAG-2 Proteins in Rodent Sertoli Cells
Source: Int J Mol Sci. 2022 Feb 18;23(4):2284. doi: 10.3390/ijms23042284 (PMC8876387; doi:10.3390/ijms23042284)
Supplement: Supplementary file 1 [file ijms-23-02284-s001.zip › ijms-1588543-supplementary.pdf]

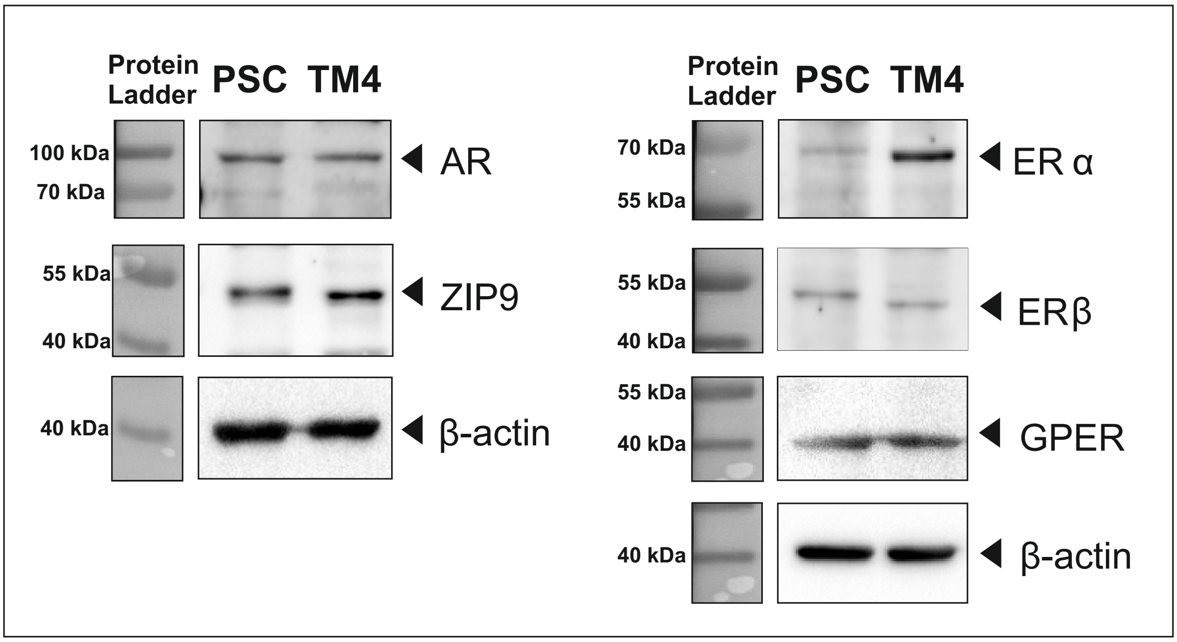

Figure S1: The expression of androgen and estrogen receptors in PSC and TM4 cells. Western blot detection of the proteins was performed as described in Material & methods section. The following primary antibodies were used: anti-AR (Cat. no. Ab74272, Sigma-Aldrich, 1:500); anti-ZIP9 (Cat. no. SAB3500599, Sigma-Aldrich, 1:1000); anti-ERα (Cat. no. Ab3575, Sigma-Aldrich, 1:1000); anti-ERβ (Cat. no. PA1-310B, Thermo Fisher Scientific, 1:1000); anti-GPER (Cat. no. Ab39742, Sigma-Aldrich, 1:1000) and anti-β-actin (Cat. no. A2228, Sigma-Aldrich, 1:3000). Electrophoretic separation of the standard proteins was carried out on the same gel as separation of the samples, but imaging of the protein ladders was performed independently after colorimetric detection.
